# Supplementary material for: Control of feeding by a bottom-up midbrain-subthalamic pathway
Source: Nat Commun. 2024 Mar 7;15:2111. doi: 10.1038/s41467-024-46430-5 (PMC10920831; doi:10.1038/s41467-024-46430-5)
Supplement: Supplementary file 1 — Supplementary Information [file 41467_2024_46430_MOESM1_ESM.pdf]

Supplementary Information

Control of feeding by a bottom-up midbrain-subthalamic pathway

Fernando MCV Reis[1]*, Sandra Maesta-Pereira[1], Matthias Ollivier[2], Peter J Schuette[1], Ekayana Sethi[1], Blake A Miranda[1], Emily Iniguez[1], Meghmik Chakerian[1], Eric Vaughn[3], Megha Sehgal[4], Darren CT Nguyen[1], Faith TH Yuan[1], Anita Torossian[1], Juliane M Ikebara[5], Alexandre H Kihara[5], Alcino J Silva[1,4,6], Jonathan C Kao[7], Baljit S Khakh[2], Avishek Adhikari[1]*

[1] Department of Psychology, University of California, Los Angeles, Los Angeles, CA, 90095, USA
[2] Department of Physiology, University of California, Los Angeles, Los Angeles, CA 90095, USA
[3] Department of Molecular and Cellular Biology, Harvard University, Cambridge, MA 02138, USA
[4] Department of Neurobiology, University of California, Los Angeles, Los Angeles, USA
[5] Centro de Matemática, Computação e Cognição, Universidade Federal do ABC, São Bernardo do Campo, SP, 09606-070, Brazil
[6] Department of Psychiatry & Biobehavioral Sciences, University of California, Los Angeles, Los Angeles, United States
[7] Department of Electrical and Computer Engineering, University of California, Los Angeles, Los Angeles, CA, 90095, USA.
*Correspondence: avi@psych.ucla.edu (A.A.); freis@ucla.edu (F.M.C.V.R.)

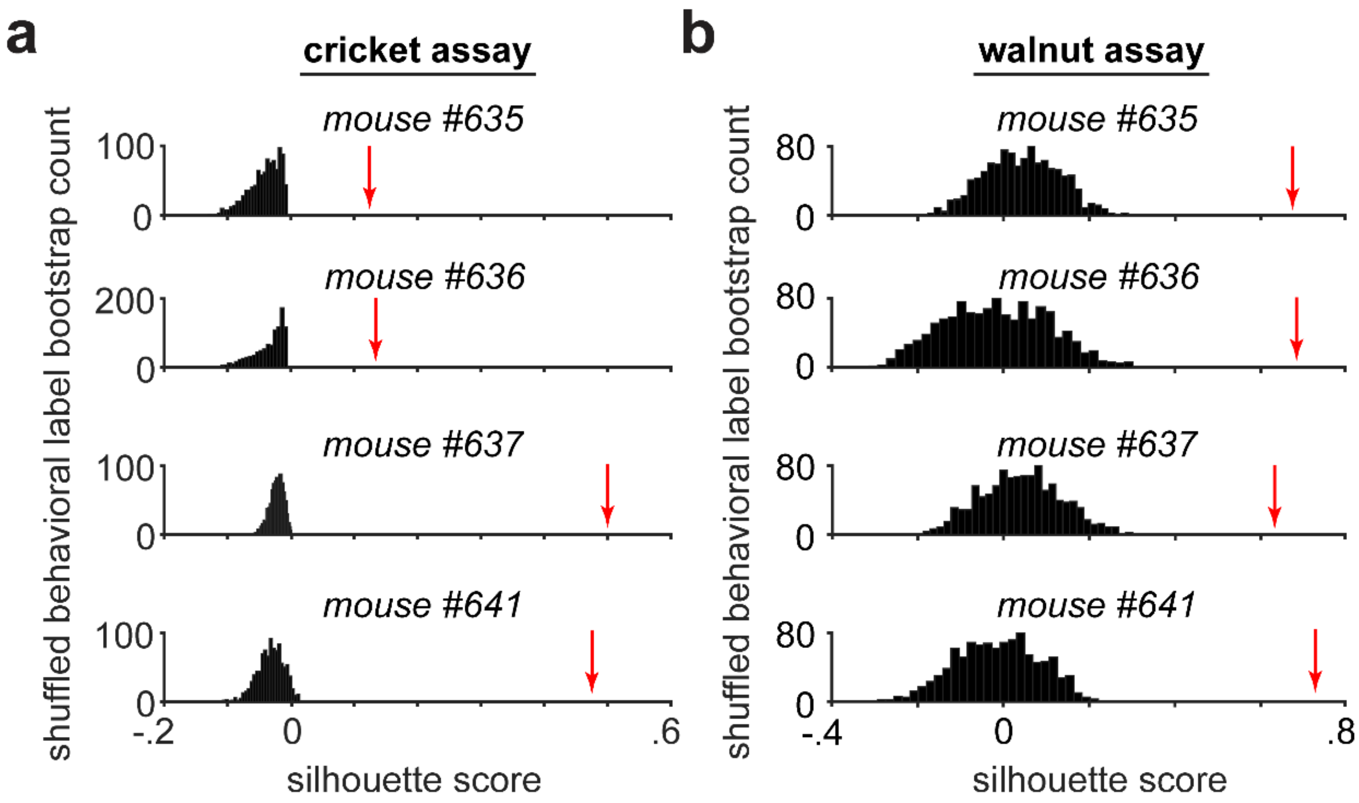

Supplementary Fig. 1: Silhouette score bootstrap for all sessions. a In order to determine if the silhouette score was significantly greater than chance for each cricket session, the behavioral labels were randomly shuffled and the silhouette score calculated over 1000 iterations, resulting in a permuted distribution, depicted here as histograms. If the actual silhouette value, depicted by a red arrow, was greater than 95% of this permuted distribution, the silhouette score was considered to be significantly greater than chance. **b** Same as **(a)**, but for the walnut assay.

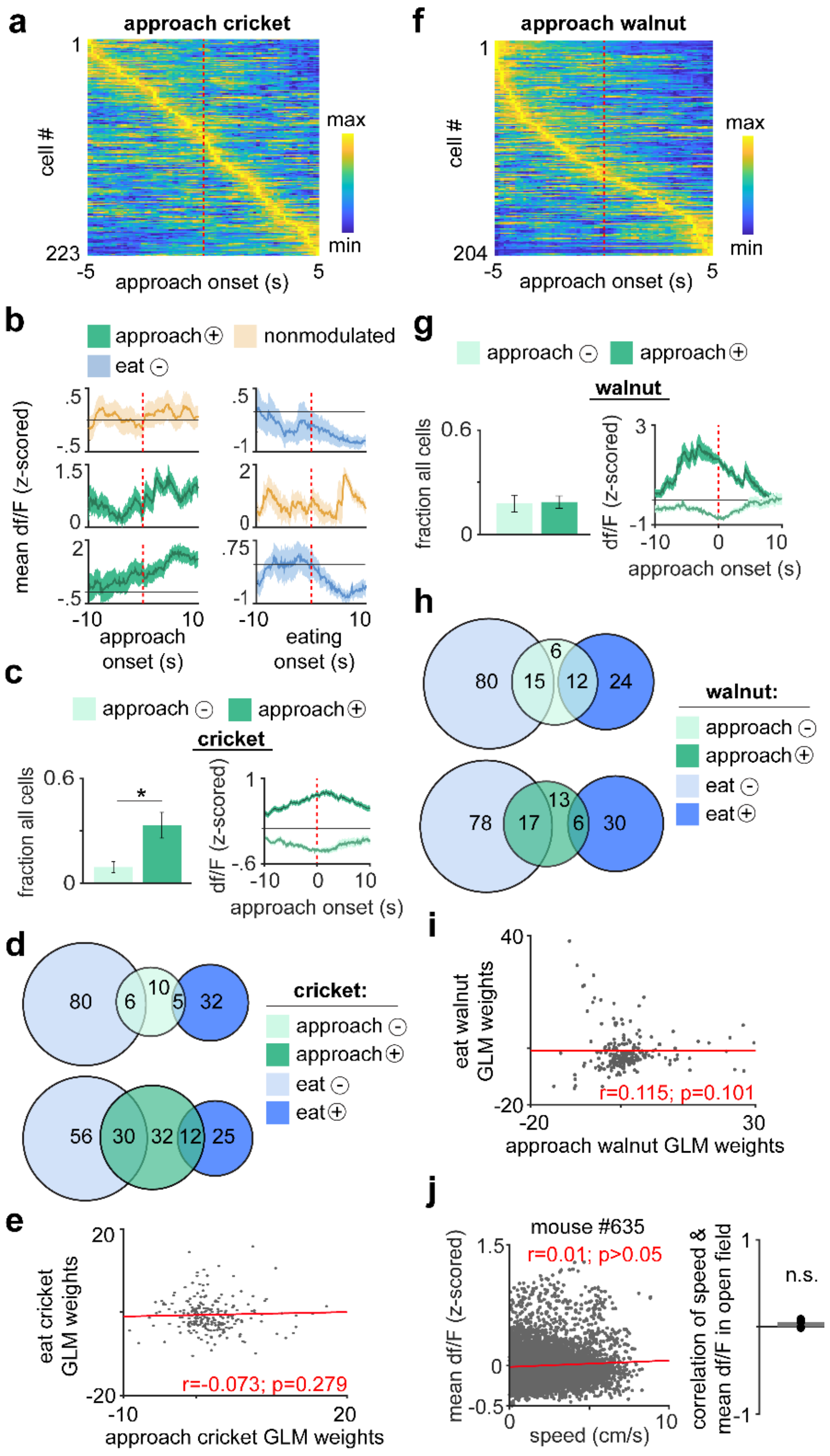

Supplementary Fig. 2: Characterization of overlap between approach and eating- modulated l/vlPAG vgat cells. a Colormap shows the activation patterns of l/vlPAG vgat cells centered on approach onset for the cricket assay (n=223). **b** Each trace depicts the mean activity of an example cell

(± 1 SEM). Activity from the same cell is plotted across each row, either centered on approach or eating onset. Cells showed a diversity of behavioral encoding; for example, some were non modulated by approach and negatively by eating (top row), others were positively modulated by approach and non-modulated by eating (middle row), still others were positively modulated by approach and negatively modulated by eating (bottom row). **c** Left: A greater fraction of cells was positively modulated by approach (left) in the cricket assay (n=4; two-sample t-test, t-statistic=-3.00). Right: Shown are mean traces (± 1 SEM) centered at approach onset for all cells that were negatively or positively modulated by approach in the cricket assay (approach - n=74, approach + n=21). **d** Venn diagrams depict the number and overlap of behaviorally classified cells in the cricket assay. **e** Scatterplot shows the correlation of eating and approach GLM weights for the cricket assay. (n=223; Spearman correlation). **f** Colormap shows the activation patterns of l/vlPAG vgat cells centered around approach onset for the walnut assay. (n=204). **g** Left: There is no significant difference in the number of cells that were positively and negatively modulated by approach in the walnut assay (n=4). Right: Shown are mean traces (± 1 SEM) centered at approach onset for cells negatively and positively modulated by approach in the walnut assay. **h** Venn diagrams depicts the number and overlap of behaviorally classified cells in the walnut assay. **i** Scatterplot depicts the correlation of eating and approach GLM weights for the walnut assay. (n=204; Spearman correlation). **j** Left: Correlation of speed and df/F (z-scored) for an example open field session. Speed was not significantly correlated with calcium activity. Right: The correlation of speed and df/F is not significantly different from zero for animals in the open field. (n=4; Wilcoxon signed rank test). *p<0.05. Source data is in the Source Data File.

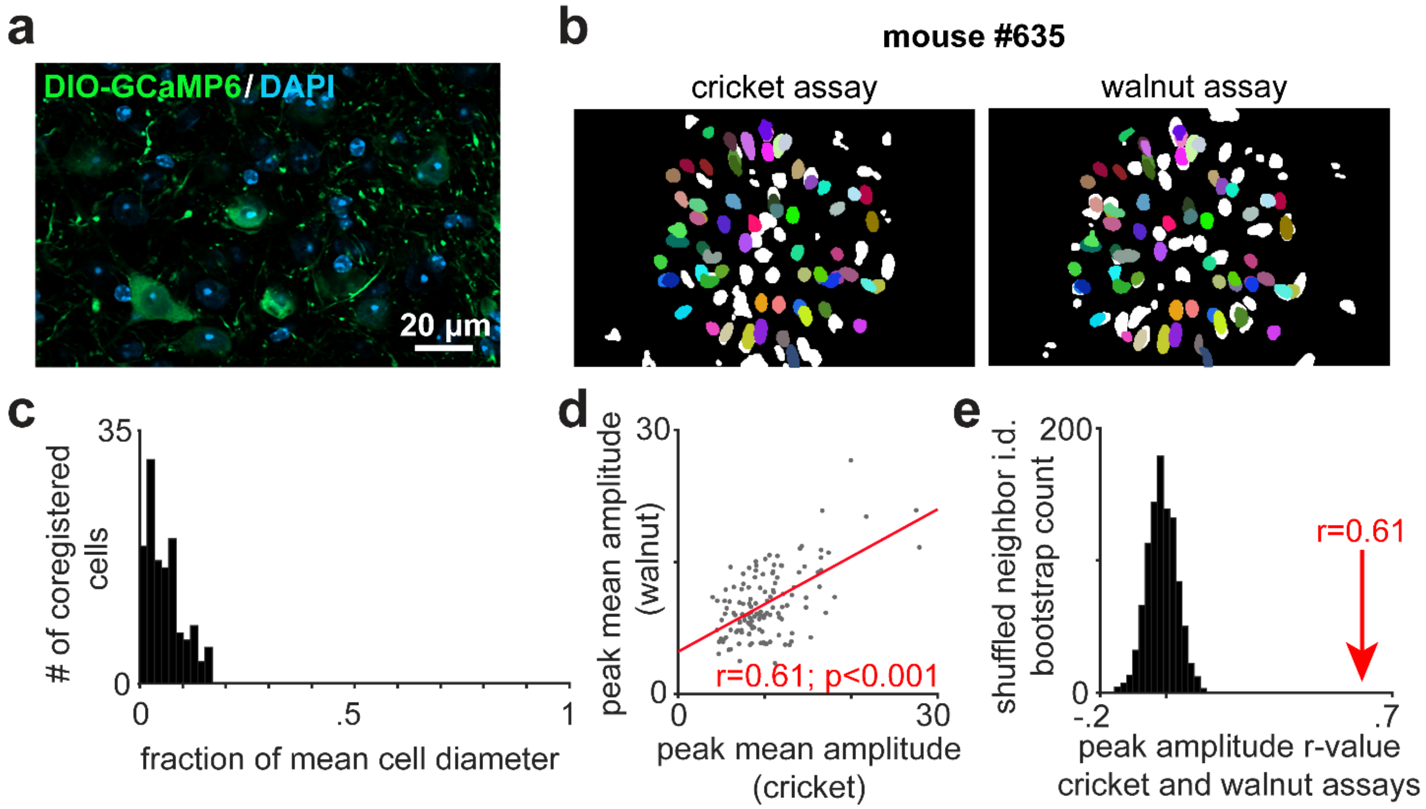

Supplementary Fig. 3: Validation for co-registration procedure across cricket and walnut assays. a Image shows expression of GCaMP6s in vgat l/vlPAG cells. **b** Example spatial contours of vgat cells identified in the same mouse for both cricket and walnut assays. White contours represent cells that were found only in one session (non-coregistered), while colorful contours represent cells that were co-registered across sessions. **c** Bars represent the displacement of coregistered cells between cricket and walnut assays as a fraction of the mean neuron diameter (n=132). **d** Shown is the peak mean amplitude for coregistered cells across cricket and walnut assays. Note these values are strongly correlated. (n=132; Spearman correlation). **e** The mean peak amplitude correlation values were calculated for coregistered cells between cricket and walnut assays, as shown in **(c)**. Cell identities were then shuffled within the 10 nearest neighbors 1000 times, and the same correlation measures were calculated for each iteration. The resulting bootstrap distribution was compared with the actual mean peak amplitude value, indicated with a red arrow (n=132; Spearman correlation; p<0.001). Source data is in the Source Data File.

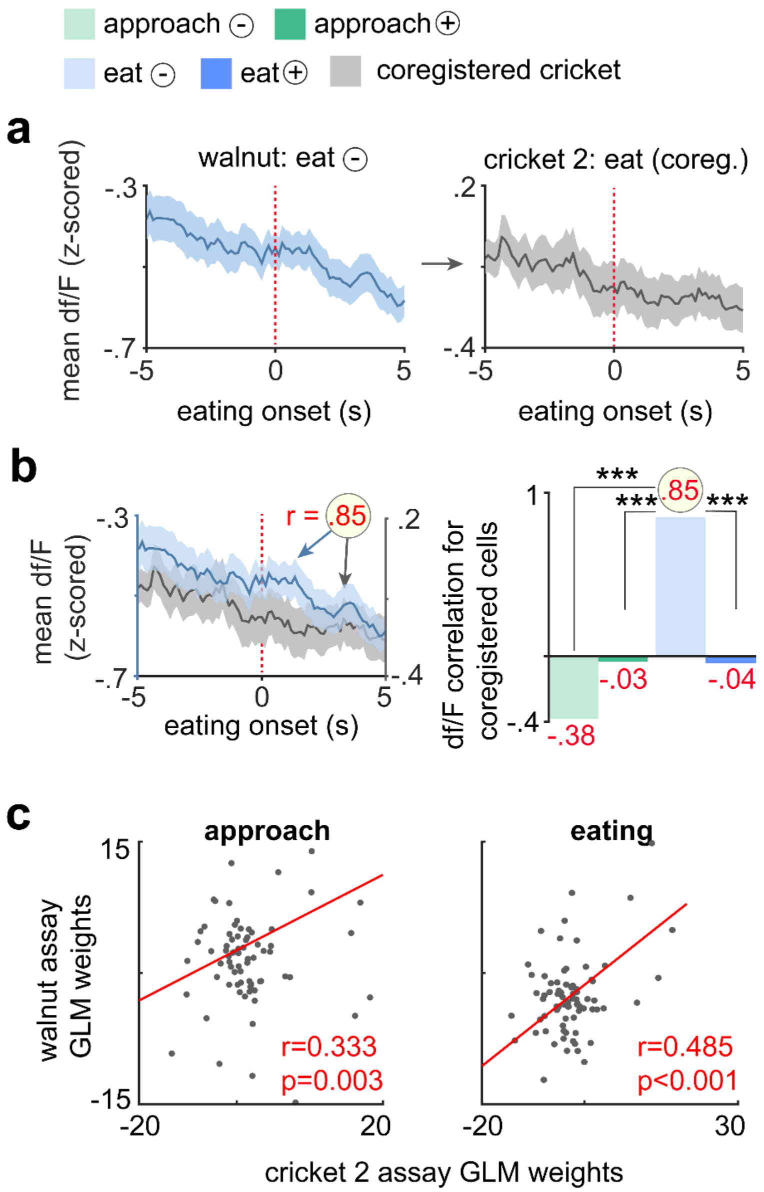

Supplementary Fig. 4: Activity of co-registered eating-inhibited l/vlPAG vgat cells is conserved across walnut and cricket assays. a Shown are the mean z-scored calcium traces (± 1 SEM) of eat - cells that coregistered across walnut and cricket 2 assays (left), as well as the activity of the same cells during the walnut assay (right), centered at eating onset (n=54). **b** Left: The coregistered traces from **(a)** have been superimposed; their mean traces are highly correlated. (Spearman correlation) Right: Bars depict the correlation between coregistered approach and eat, positively and negatively classified cells for walnut and cricket 2 assays. Eat - cells exhibit significantly greater correlation across assays than the other three cell types. (approach - n=12, approach + n=20, eat - n=54, eat + n=12; Spearman correlation; for r-value comparison, Fisher r-to-z transformation). **c** Correlation of approach (left) and eating (right) GLM weights for coregistered cells across the walnut and cricket 2 assays. The weights are significantly correlated for both behaviors. (n=78; Spearman correlation). ***p<0.001. Source data is in the Source Data File.

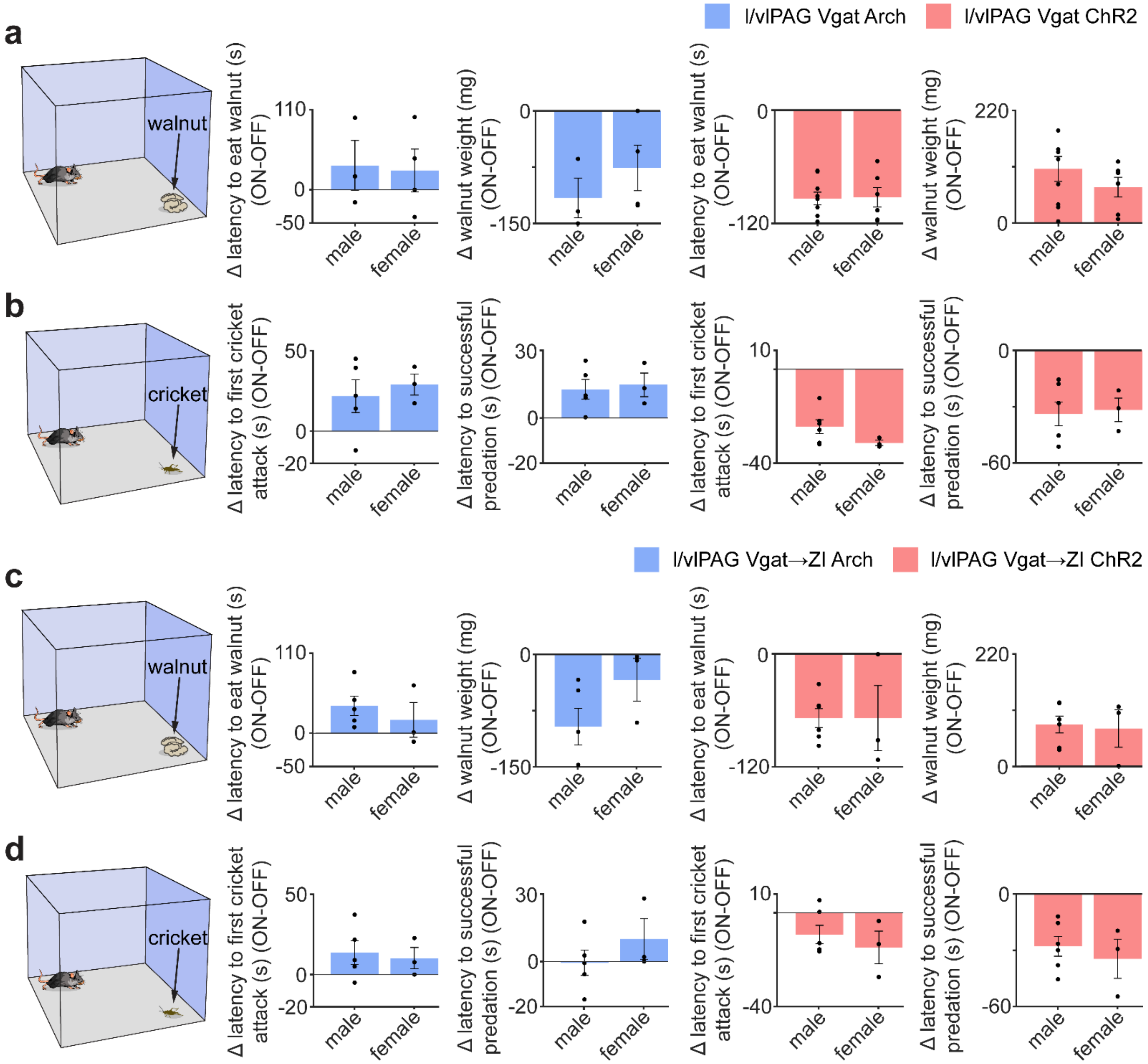

Supplementary Fig. 5: Male and female mice did not show any behavioral differences during excitation or inhibition of l/vlPAG vgat cells in cricket and walnut assays. a (left) Scheme of the walnut assay. (right) Bars compare key metrics from this assay across male and female Vgat Arch (blue) and ChR2 (red) mice, and conclude the difference is nonsignificant. (Wilcoxon rank-sum test; Walnut assay: Arch male n=3, Arch female n=4; ChR2 male n=9, ChR2 female n=6; Cricket assay: Arch male n=5, Arch female n=3; ChR2 male n=6, ChR2 female n=3) **b** Same as **a**, but for the cricket assay. **c-d** Same as **a-b**, but for l/vlPAG vgat→ZI (Arch) and l/vlPAG vgat→ZI (ChR2) mice. (Wilcoxon rank-sum test; Walnut assay: Arch male n=5, Arch female n=3; ChR2 male n=6, ChR2 female n=3; Cricket assay: Arch male n=5, Arch female n=3; ChR2 male n=6, ChR2 female n=3). Source data is in the Source Data File.

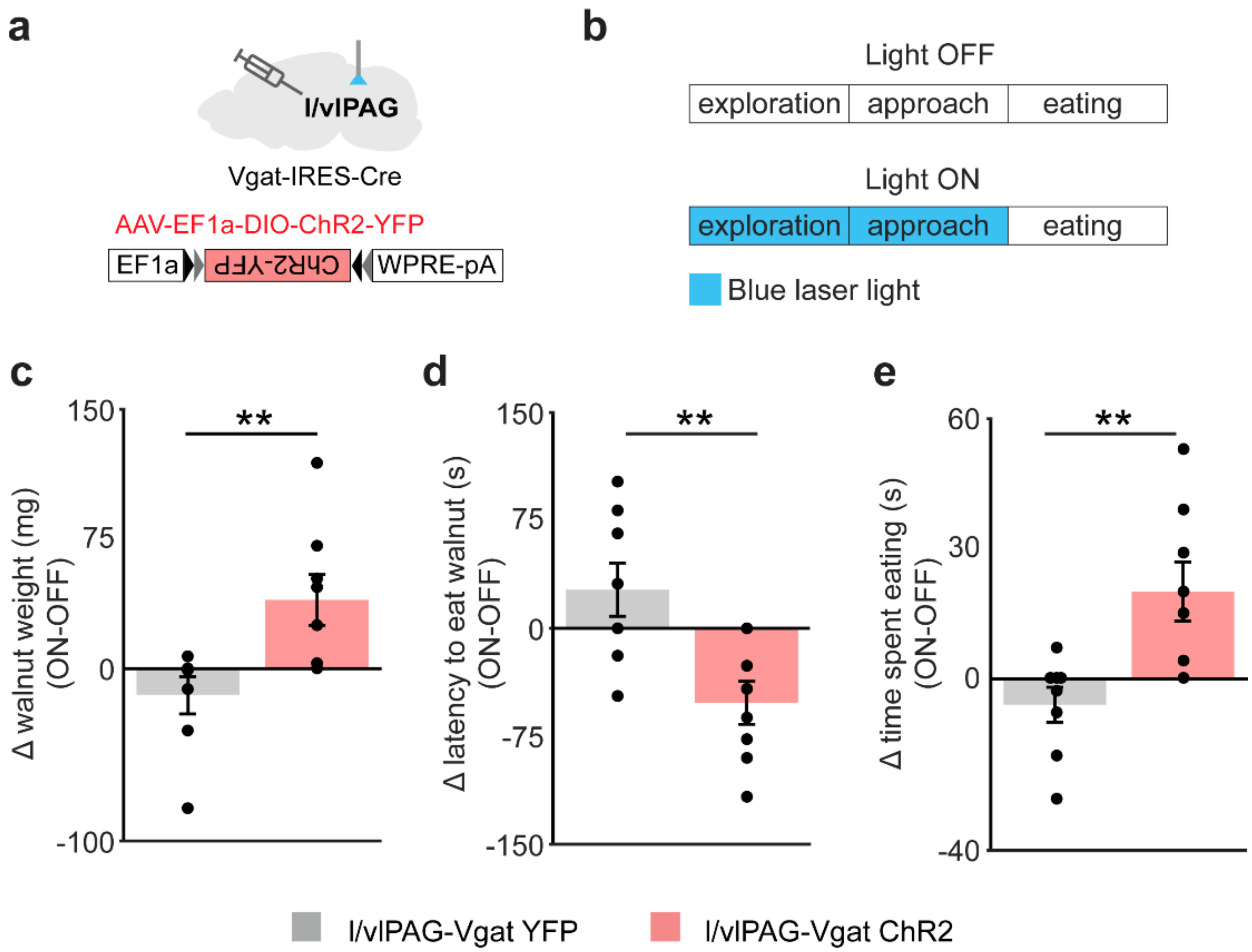

Supplementary Fig. 6: Optogenetic activation of l/vlPAG vgat cells only during approach to food increases food consumption. a Scheme showing vector used to express ChR2 in l/vlPAG vgat cells. **b.** Optogenetic excitation with blue light was only delivered during the ON epoch during exploration and approach. Blue light was not delivered during consumption to emulate the endogenous neural activity of these cells, as these cells show lower activity during consumption (see Fig. 2). Optogenetic excitation of l/vlPAG vgat cells induced consumption (**c**), decreased the latency to eat (**d**) and increased the amount of time spent eating (**e**). (YFP n=8, ChR2 n=8, left: Wilcoxon rank-sum test p<0.009, 0.004 and 0.004, respectively for food eaten, latency and time spent plots). Source data is in the Source Data File.

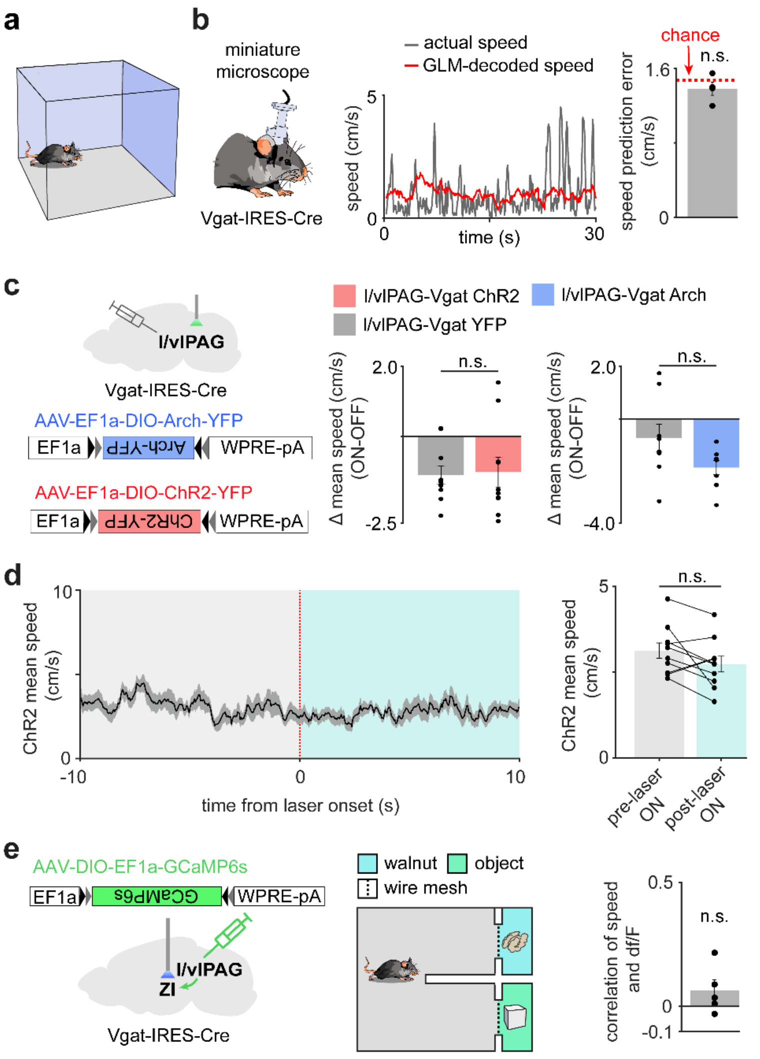

Supplementary Fig. 7: L/vlPAG vgat cells do not affect overall locomotion. (a) Scheme of open field assay. **(b)** A generalized linear model was trained with miniscope-recorded neural data (predictor variable) and mouse speed in the open field assay (response variable). The model was then used to predict speed, given a withheld test dataset, and performed at a level no better than chance (Wilcoxon signed rank test; n=4). **(c)** Optogenetic excitation or inhibition of l/vlPAG vgat cells did not alter speed (ChR2 experiment: mCherry n=10, ChR2 n=10; Arch experiment: mCherry n=8, Arch=9; Wilcoxon rank-sum test). **(d)** The trace on the left shows the mean speed (+/- SEM) of ChR2 mice in the open field assay, centered at laser onset. The speed pre- and post-laser onset were not significantly different (Wilcoxon signed rank test; n=10) **(e)** (left) Vgat-Cre mice were injected with a vector encoding cre-dependent GCaMP6s in the l/vlPAG. A fiberoptic cannula was implanted above the zona incerta (ZI) to record calcium transients in l/vlPAG vgat axon terminals in the zona incerta. (middle) Mice were exposed to the environment depicted in the scheme. Mice were able to see and smell the walnut through the wire mesh but were not able to eat or touch it. (right) The recorded fiber photometry signal was not significantly correlated with mouse speed (Wilcoxon signed rank test; n=5). Source data is in the Source Data File.

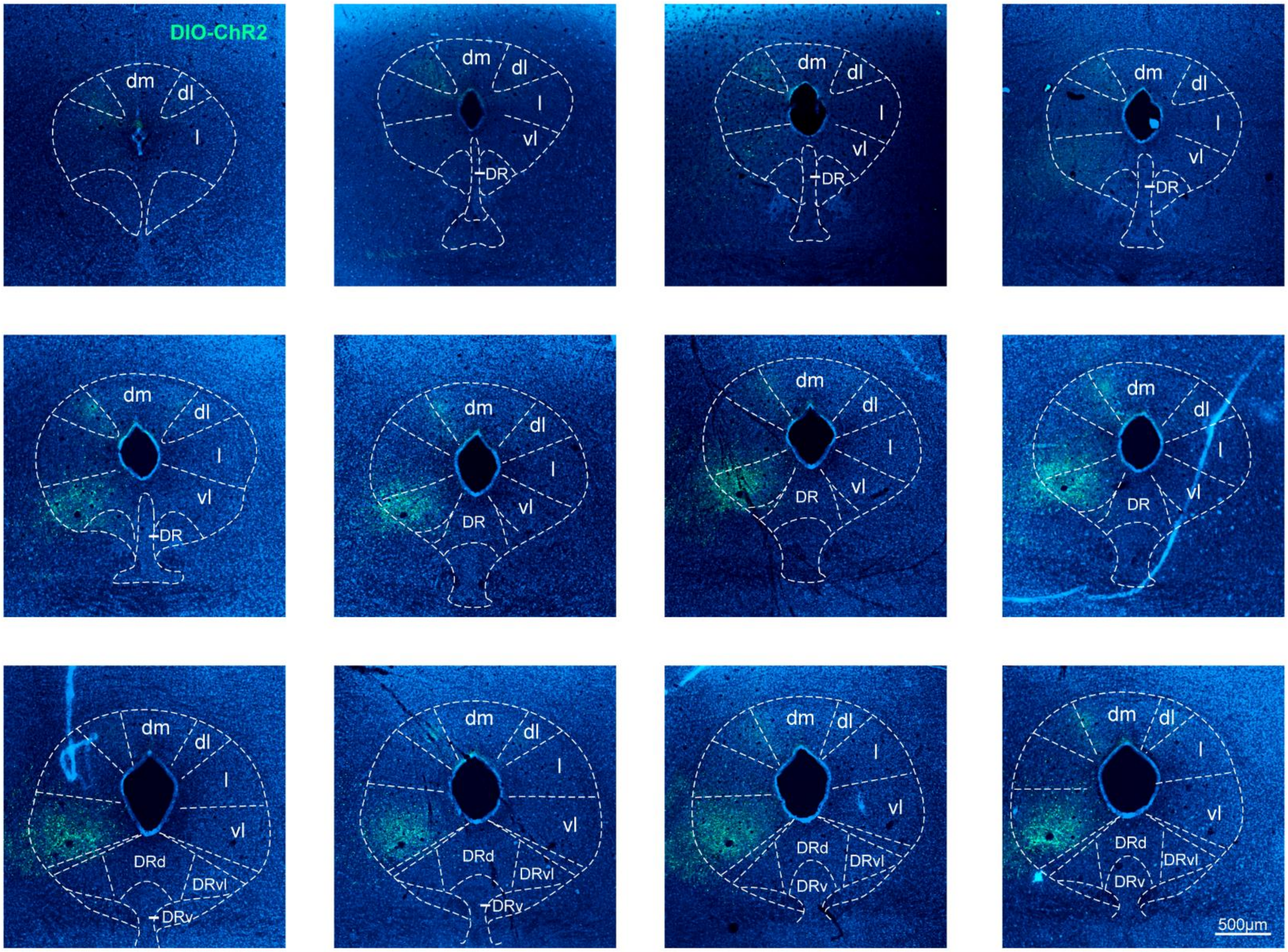

Supplementary Fig. 8: Characterization of viral spread throughout the anteroposterior axis shows expression is restricted to the l/vlPAG. Expression of DIO-ChR2 in vgat l/vlPAG cells throughout the anterior posterior axis of the PAG. Representative serial images from a single mouse showing expression of YFP (green) restricted to the l/vlPAG.

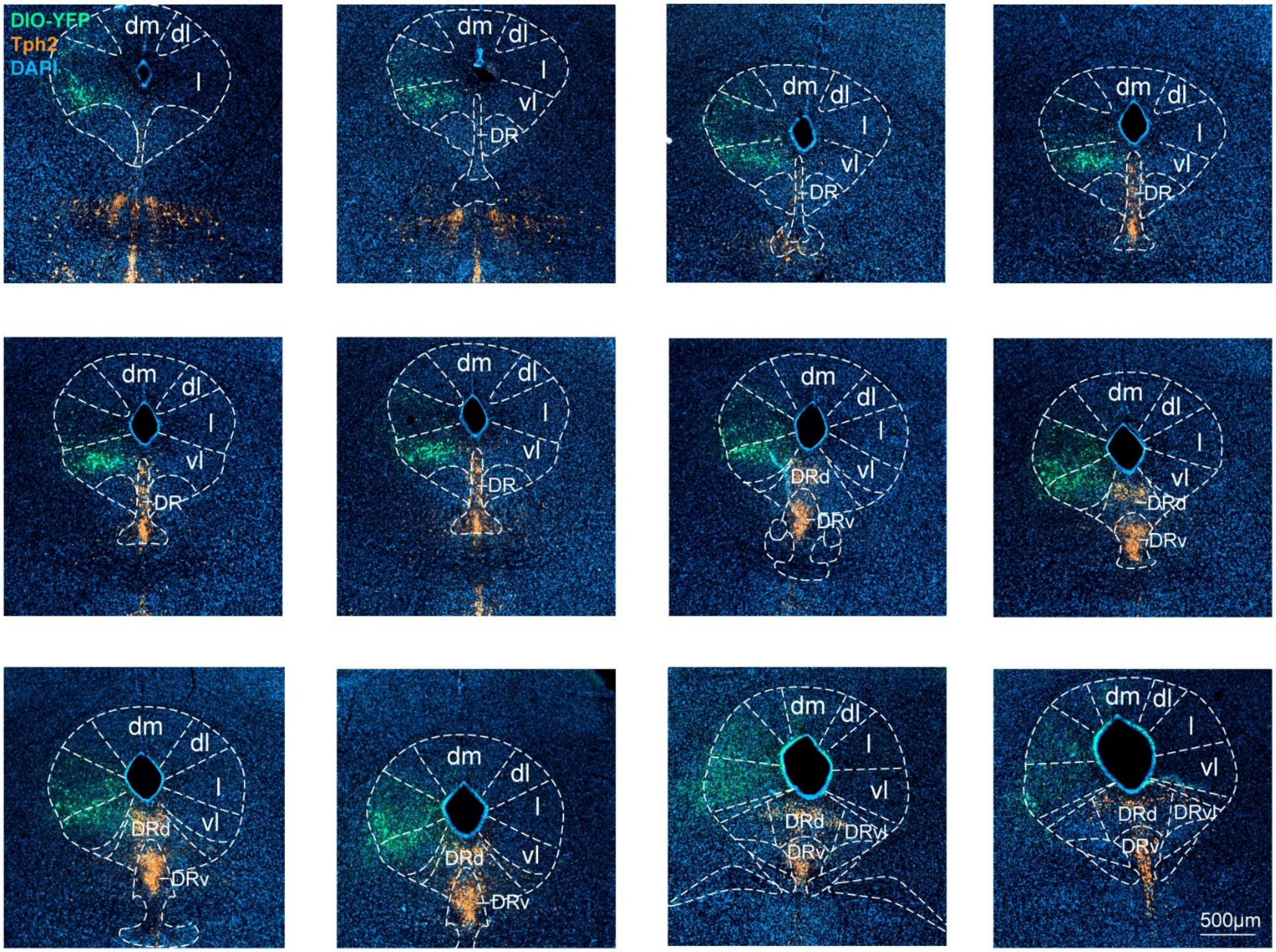

Supplementary Fig. 9: Viral expression was restricted to the l/vlPAG and did not spread to the dorsal raphe. Viral vector spread was restricted to the l/vlPAG throughout the anterior posterior axis of the PAG and did not spread over into the dorsal raphe. Representative images from a single mouse showing that expression of YFP in l/vlPAG cells (green) was contained within the boundaries of the PAG. The outline of the dorsal raphe is made visible by tyrosine hydroxylase 2 (Tph2) - expressing cells (orange). Note that the green YFP-expressing region shows no overlap with the orange dorsal raphe region throughout a large range of the anterior-posterior axis.

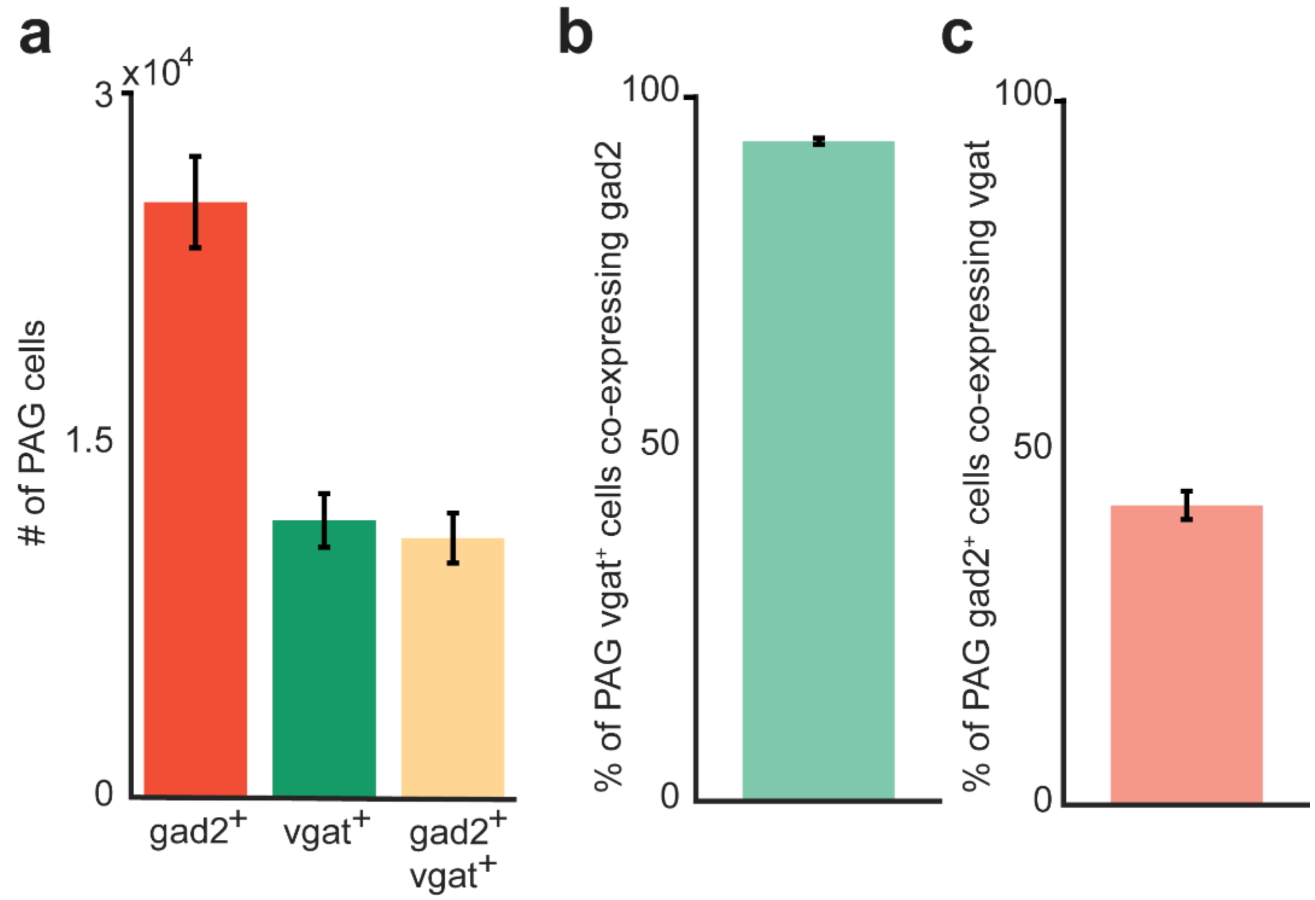

Supplementary Fig. 10: The majority of PAG gad2-expressing cells do not co-express vgat. (a) Analysis of the MERFISH data from Vaughn et al quantifying the number of gad2, vgat and gad2*vgat expressing cells in the PAG per mouse. (b) Note that the vast majority (92%) of vgat cells co-express gad2. (c) Graph showing that 42% of gad2-expressing cells also co-express vgat. Data are plotted as mean (+/- SEM). n=784,863 gad2+, 366,011 vgat+, 342,886 vgat+.gad2+, n=31 mice. Source data is in the Source Data File.

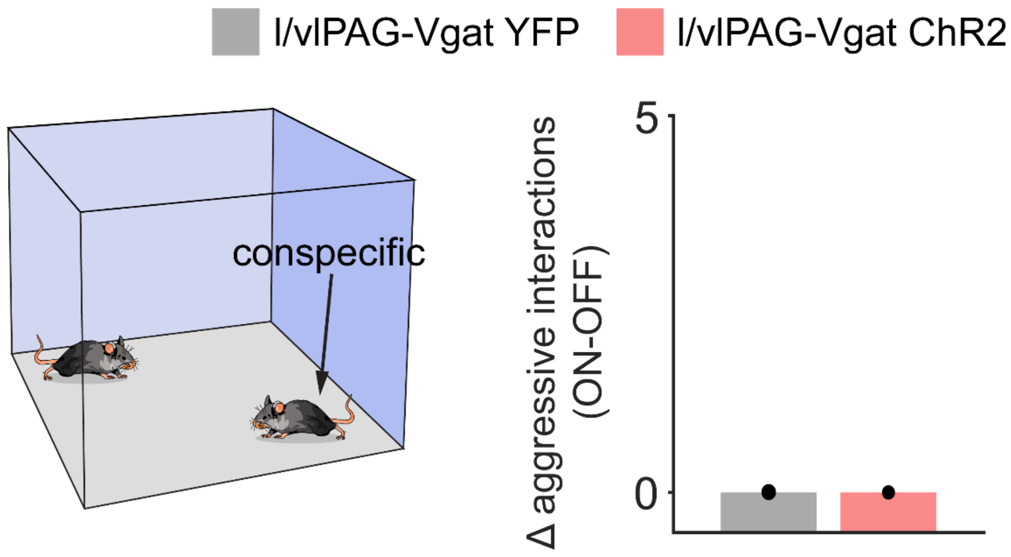

Supplementary Fig. 11: Optogenetic stimulation of l/vlPAG vgat cells did not induce biting or aggression towards another mouse. Delivery of blue light in ChR2-expressing l/vlPAG vgat cells did not induce aggression or biting of a conspecific. (YFP n=6, ChR2 n=5). Source data is in the Source Data File.

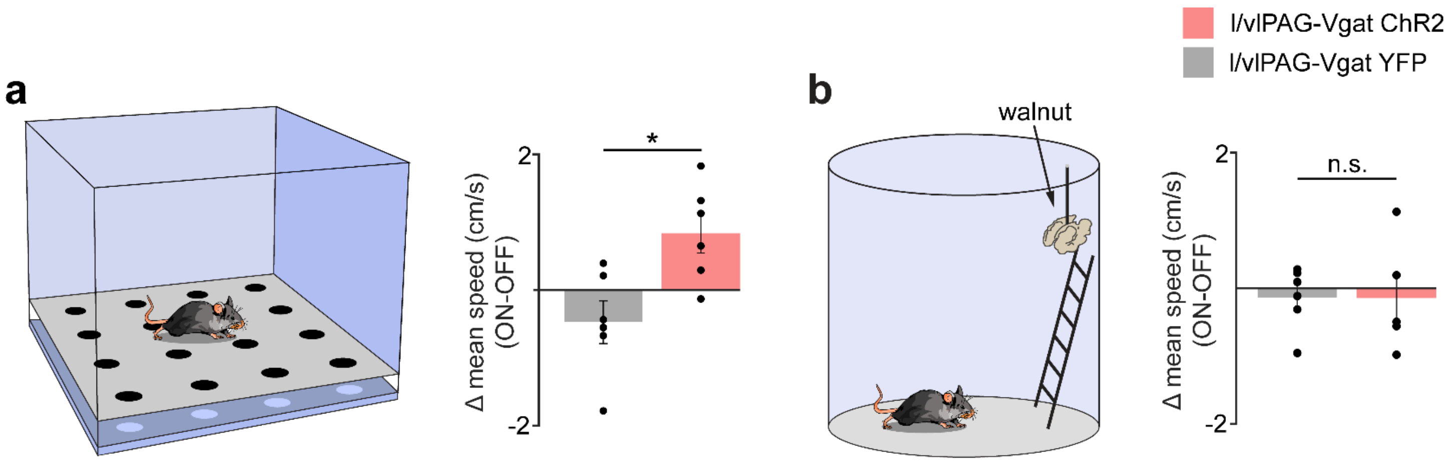

Supplementary Fig. 12: Activation of l/vlPAG vgat cells increases speed in the holeboard task, but not in the hanging walnut test. a Activation of l/vlPAG vgat cells increased the mean speed in the hole board test. (YFP n=6, ChR2 n=6, Wilcoxon rank-sum test, z-value=-2.487, *p=0.015). **b** Bars depict the difference in mean speed (ON-OFF) in the hanging walnut assay. (Wilcoxon rank-sum test; YFP n=6, ChR2 n=5, Wilcoxon rank-sum test, z-value=0.437, p=0.662). Source data is in the Source Data File.

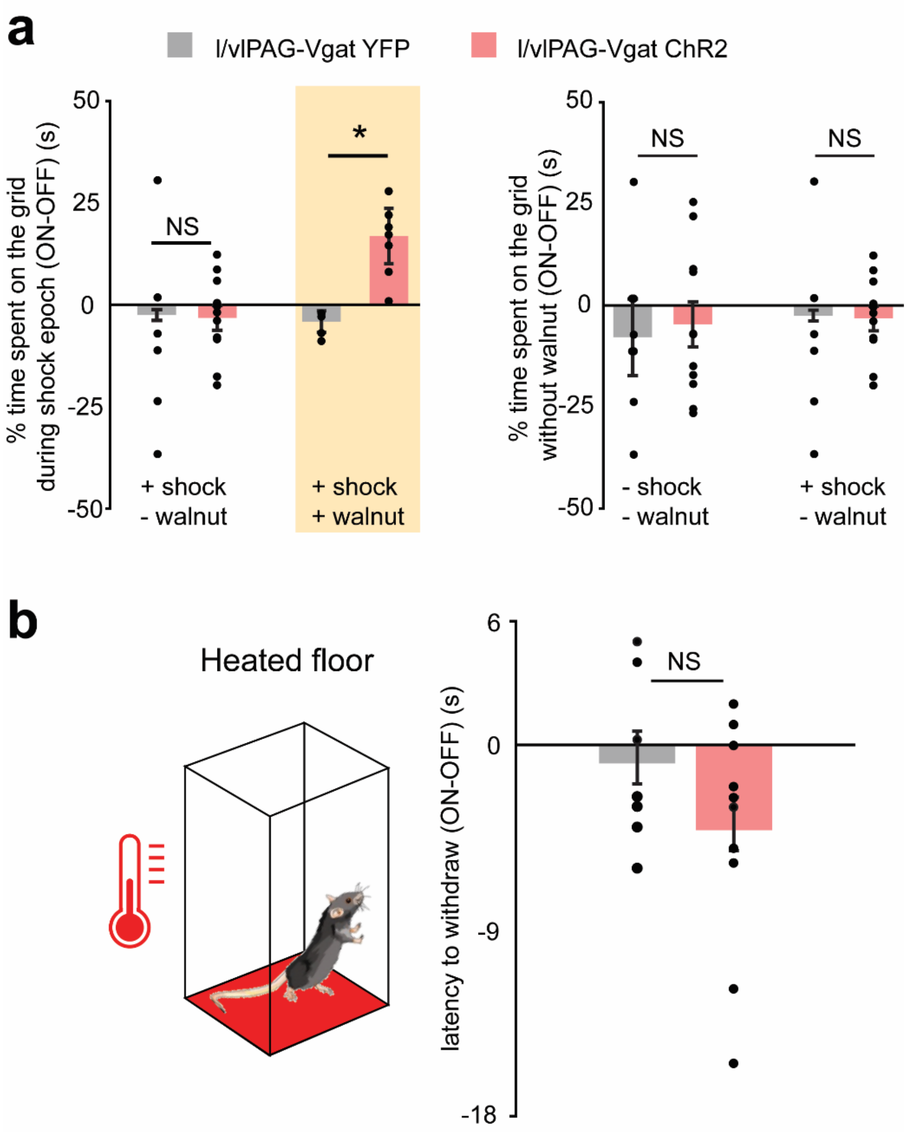

Supplementary Fig. 13: Optogenetic activation of l/vlPAG vgat cells increases time on the shock grid only in the presence of walnut reward. a. Mice were exposed to an environment with a safe floor and a shock grid floor. In the presence of shock, but absence of walnut (first two bars) both YFP and ChR2 mice avoid the shock grid to similar extent, indicating that activation of l/vlPAG vgat cells does not decrease pain sensitivity. We then added walnut reward to the shock grid, and observed that activation of ChR2-expressing l/vlPAG vgat cells increases time spent on the grid (yellow rectangle) (Wilcoxon rank-sum test; *p<0.05, n=5 YFP, n=7 ChR2). **b.** Optogenetic excitation of l/vlPAG vgat cells did not change latency for nociceptive reactions in the heated floor assay, showing that activation of l/vlPAG vgat cells does not change pain reactions in the hot plate assay. Graph depicting average latency for pain reactions in the heated floor assay for mice expressing either YFP or ChR2 in l/vlPAG vgat cells (Wilcoxon rank-sum test; p>0.05, YFP n=7, ChR2 n=11). Source data is in the Source Data File.

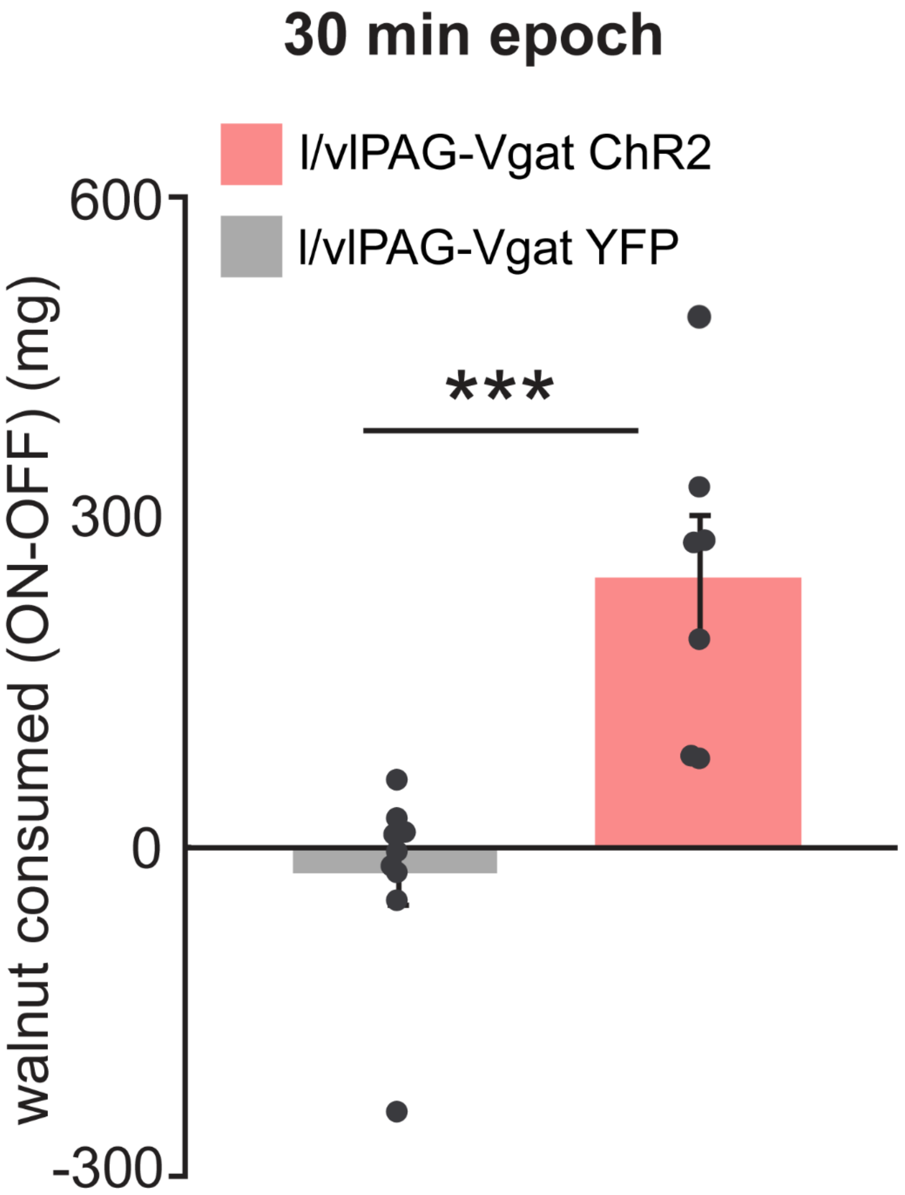

Supplementary Fig. 14: Optogenetic activation of l/vlPAG vgat cells for longer times also increases food consumption. Mice expressing either ChR2 or YFP in l/vlPAG vgat cells were exposed to walnuts for 60 minutes. Optogenetic excitation happened during the 2nd half (minutes 30 to 60) of the assay. Data are plotted as mean +/- SEM of walnut consumed. n=7 ChR2, n=9 YFP, Wilcoxon rank-sum test. *** p<0.001. Source data is in the Source Data File.

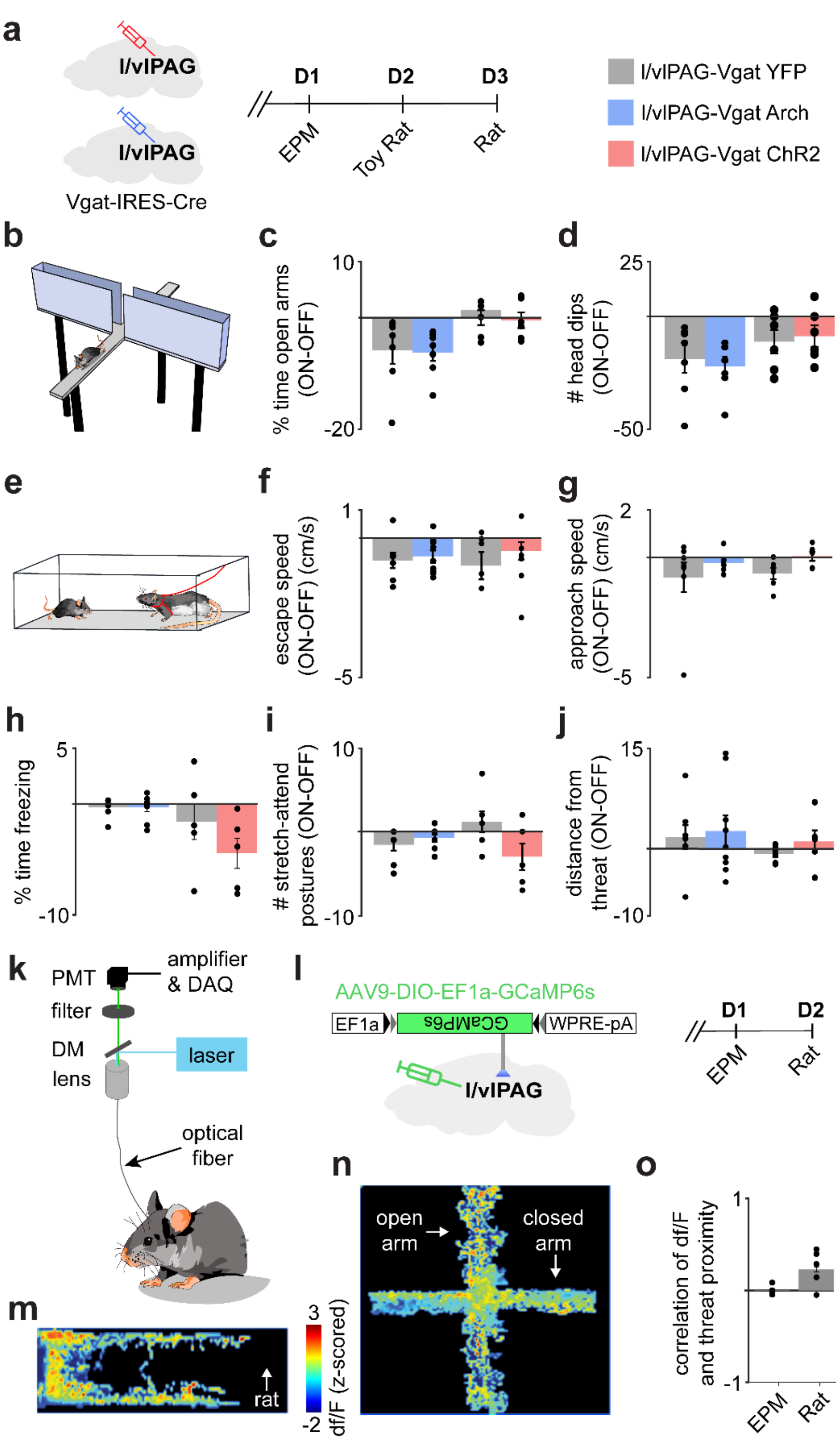

Supplementary Fig. 15: Vgat l/vlPAG cell activity is not necessary or sufficient to control innate defensive behaviors. a Vgat-cre mice were injected with viral vectors to express either YFP, Arch or ChR2 in l/vlPAG vgat cells. (**b-d**) Optogenetic activation or inhibition of l/vlPAG vgat cells did not alter exploration of the open arms or the number of head dips in the EPM. (EPM with Arch: Arch n=7, YFP n=7; EPM with ChR2: ChR2 n=5, YFP n=6). **e** Scheme of the rat assay. Optogenetic activation or inhibition of vgat l/vlPAG cells did not alter escape speed (**f**), approach speed (**g**), freezing (**h**), stretch-attend postures (**i**) or distance from the rat (**j**). (Rat assay with Arch: Arch n=9, YFP=7; Rat assay with ChR2: ChR2 n=5, YFP n=6; EPM with Arch: Arch n=6, YFP n=6; EPM with ChR2: ChR2 n=6, YFP n=6). **k** Scheme showing fiber photometry setup to collect data from GCaMP6s-expressing l/vlPAG vgat cells. **l** A cre-dependent viral vector was used to express the calcium indicator GCaMP6s in l/vlPAG vgat-expressing cells (left). The order of behavioral assays is shown on the right. **m** Representative neural activity heat map in the rat assay, showing that vgat cells are not more activated near the rat (rat location is indicated by the white arrow). **n** Heat map of neural activity in the elevated plus maze (EPM) showing homogenous activation across arm types. **o** Correlation of neural activity with distance to threat in the EPM and rat assays. (EPM n=7, rat n=6). Source data is in the Source Data File.

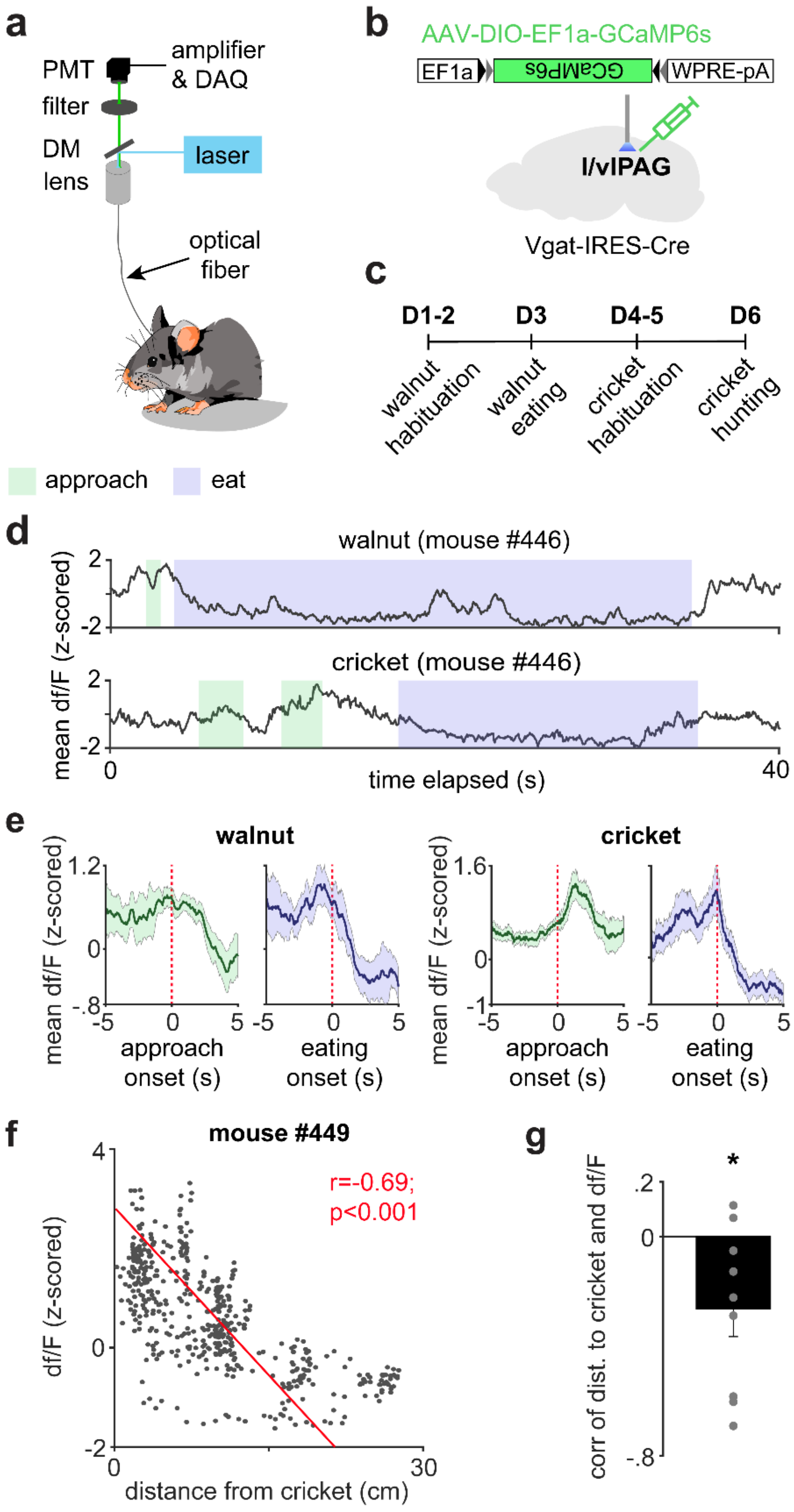

Supplementary Fig. 16: L/vlPAG vgat cells encode approach, eating and distance to prey. a Scheme of fiber photometry setup. **b** The viral vector AAV-DIO-Ef1a-GCaMP6s was injected in the l/vlPAG of vgat-Cre mice. **c** Experimental timeline. **d** l/vlPAG vgat activity was elevated prior to eating during approach (green shaded area) and decreased during eating (blue shaded area). **e** Average traces of neural activity centered at approach and eating onset for walnut and cricket assays. (walnut n=6; cricket n=9). **f** Example scatter plot showing a negative correlation between l/vlPAG neural activity and the distance from cricket (samples n=642; Spearman correlation). **g** Average data showing that vgat activity and distance to cricket is negatively correlated across mice (n=9; one-sample t-test, t-statistic=-2.41). *p<0.05. Source data is in the Source Data File.

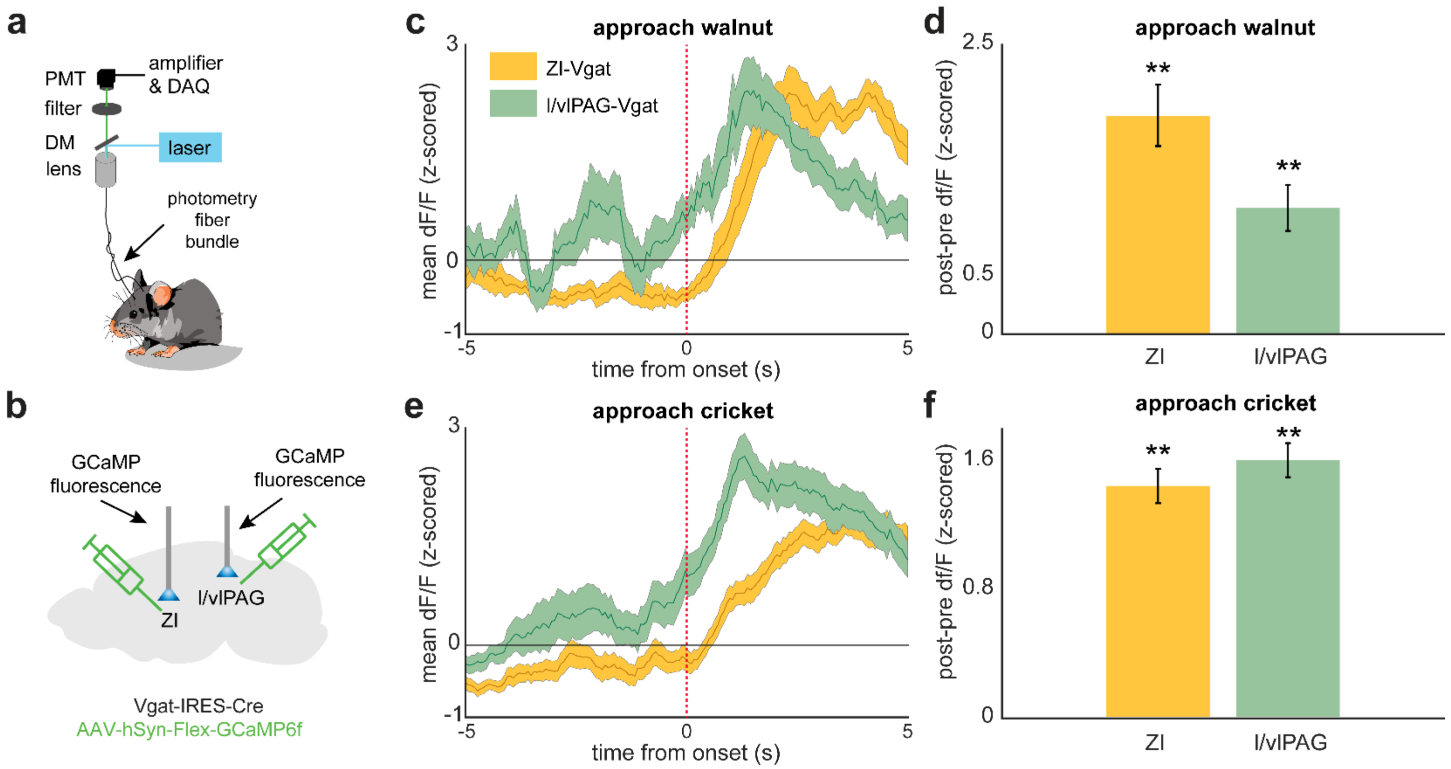

Supplementary Fig. 17: ZI and l/vlPAG cells are activated during approach to food. Dual photometry recording experiment in vgat-expressing cells from l/vlPAG and ZI. AAV-FLEX-GCaMP6f was injected in the l/vlPAG and ZI of vgat-Cre mice. Recordings of calcium transients were obtained from vgat ZI and vgat l/vlPAG cell bodies from contralateral hemispheres to avoid contamination of the signal from long range axon terminals (**a,b**) . The data show that in both regions vgat cells are activated during approach to walnut (**c,d**) and cricket (**e,f**). 3 mice, n=14 trials for walnut and n=20 trials for cricket; t-test, t-statistic (ZI-walnut)=16.56, $p<0.001$; t-statistic (l/vlPAG-walnut)=12.77, $p<0.001$; t-statistic (ZI-cricket)=26.17, $p<0.001$; t-statistic (l/vlPAG-cricket)=29.21, $p<0.001$. Source data is in the Source Data File.

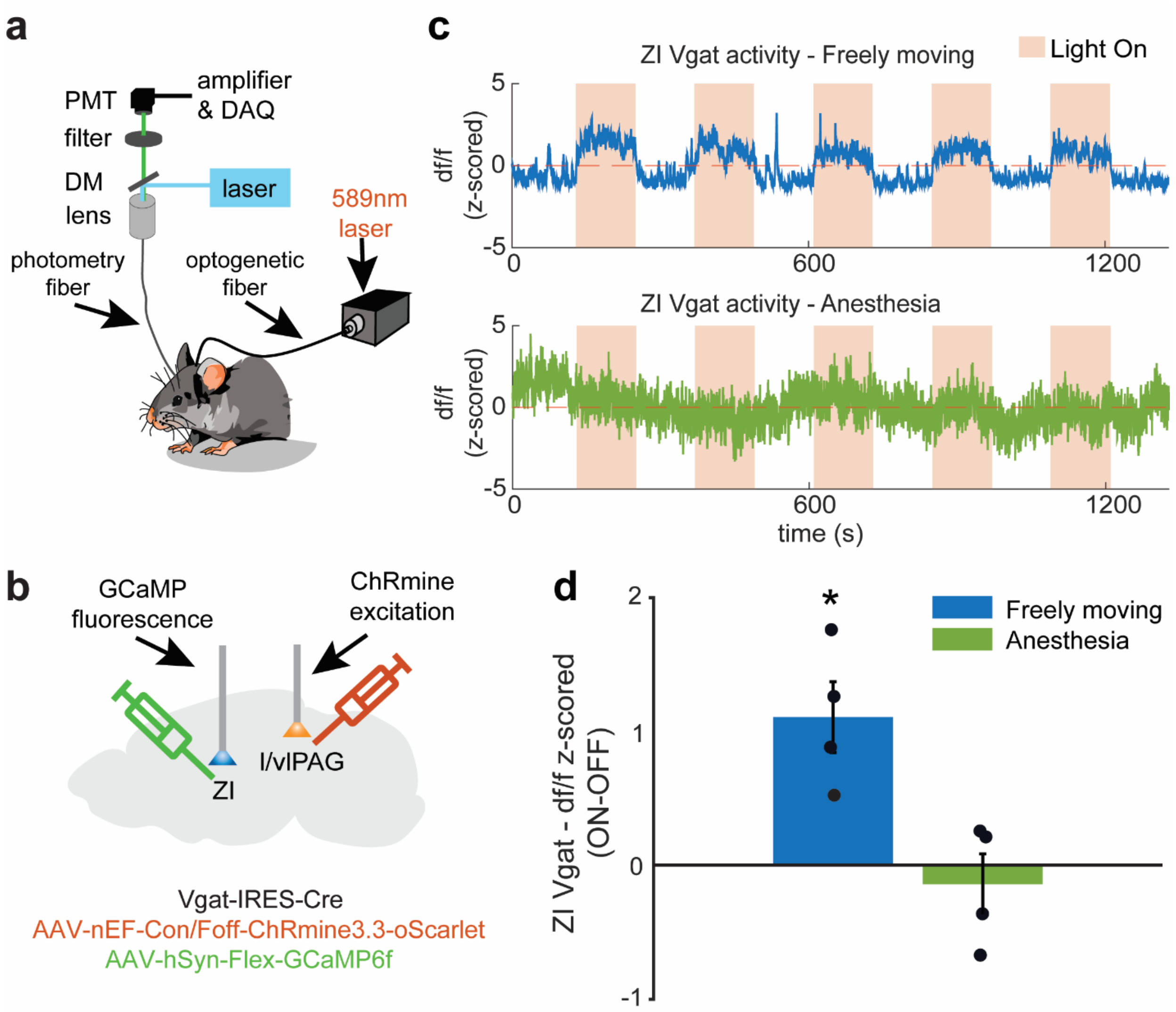

Supplementary Fig. 18: Optogenetic activation of /vlPAG vgat cells increases activity in zona incerta vgat cells. a Scheme showing mouse with simultaneous recording of GCaMP6f-expressing zona incerta vgat cells with 589 nm optogenetic excitation of ChRmine-expressing l/vlPAG vgat cells. **b** Cre-dependent viral vectors encoding GCaMP6f and the red-shifted opposing ChRmine were injected, respectively, in the zona incerta and l/vlPAG of a vgat-Cre mouse. Fiberoptic cannulae for fiber photometry and optogenetic excitation were, respectively implanted over the zona incerta and the l/vlPAG. **c** Upper panel: Activity from GCaMP6f-expressing vgat zona incerta cells in a representative freely-moving mouse with or without optogenetic stimulation of ChRmine-expressing l/vlPAG vgat cells. Stimulation epochs (light ON) are indicated by shaded orange rectangles. Lower panel: Same as upper panel, but with recordings from the same representative mouse under isoflurane anesthesia. **d** Average activity in zona incerta vgat cells (plotted as light ON - light OFF). Note that optogenetic stimulation of l/vlPAG vgat cells increases activity in zona incerta vgat cells in freely moving mice (blue), but not in isoflurane anesthetized mice (green). n=4, paired t-test, t-statistic=4.18, p-value=0.025. Source data is in the Source Data File.
