# Supplementary material for: Control of feeding by a bottom-up midbrain-subthalamic pathway
Source: Nat Commun. 2024 Mar 7;15:2111. doi: 10.1038/s41467-024-46430-5 (PMC10920831; doi:10.1038/s41467-024-46430-5)
Supplement: Supplementary file 3 — Description of Additional Supplementary Information [file 41467_2024_46430_MOESM3_ESM.pdf]

Description of Additional Supplementary Files

File Name: Supplementary Movie One

Description: Optogenetic activation of l/vlPAG vgat cells induces following of a plastic table tennis ball. The ball was moved in a trajectory that spelled the letters “BG”. Mouse and ball tracks are shown, respectively, in black and orange. Mice had no training with this assay and the ball is not coated with food.

File Name: Supplementary Movie Two

Description: Optogenetic activation of l/vlPAG vgat cells induces mice to climb a wire-mesh to eat walnuts that are suspended above the ground.

File Name: Supplementary Movie Three

Description: Optogenetic activation of l/vlPAG vgat cells induces mice to hunt live cricket prey.
